# Relationship between perceived risk and compliance with infection control measures during the first year of a pandemic

**DOI:** 10.7717/peerj.20554

**Published:** 2026-02-19

**Authors:** Sebastian B. Bjørkheim, Sigurd W. Hystad, Bjørn Sætrevik

**Affiliations:** Department for Psychosocial Science, Faculty of Psychology, University of Bergen, Bergen, Norway

**Keywords:** Perceived risk, Compliance, COVID-19, Health protective behavior, Infection control measures, Cross-lagged analysis, Registered report

## Abstract

The way people perceive health risks is often assumed to influence how they adopt precautionary measures. However, people’s assessment of a given phenomenon’s risk may vary over time, and the relationship between perceived risk and compliance with protective measures may be dynamic and bi-directional. We measured the perceived risk of COVID-19 and compliance with infection control measures for a large representative sample at four time-points during the first year of the COVID-19 pandemic in Norway. We employed a cross-lagged panel analysis to investigate both the cross-sectional and the temporal association between perceived risk and compliance. We found cross-sectional associations between perceived risk and compliance at one of the time points. There were no temporal associations between risk at one time-point and compliance at the subsequent time-point. Neither was compliance associated with risk at the subsequent time-point. The results suggest that the relationship between perceived risk and compliance with COVID-19 infection control measures is negligible and stable over time. A multiverse analysis showed that the absence of a relationship between perceived risk and compliance was robust to different operationalizations of perceived risk. This highlights the need for a nuanced understanding of how risk perceptions impact behavior during a pandemic.

## Introduction

### Background

Compliance with infection control measures may be decisive for determining the societal impact of a pandemic event. During the COVID-19 pandemic, public health authorities requested people to change their daily routines such as working from home, avoiding social gatherings, and limiting travel and the use of public transportation. People were also advised to take precautions such as wearing face masks, keeping physical distance from each other, and being careful about personal hygiene. While these measures are important to limit infection spread, their effectiveness depends on people’s willingness to comply with them.

The theory of planned behavior (Ajzen, 1985; Ajzen, 1991) posits that attitudes, subjective norms, and perceived behavioral control shape behavioral intentions, which in turn determine behavior. Research on health-protective behavior typically places perceived risk as a core reason for compliance with health recommendations (Brewer et al., 2007). Over the course of a pandemic event, motivation to comply may fluctuate as infection risk varies, fatigue sets in, and people change their opinion of the precautionary advice. Compliance over an extended period can thus be viewed as a balancing act between protecting one’s somatic health and maintaining one’s mental health. This balancing act may lead to variations in how people see pandemic risks and their compliance with infection control measures, and priorities may change over time.

#### Perceived risk

Risk can be considered the product of two main factors: the probability of something occurring and the severity of the outcome. Typically, the term “risk” is used when the outcome is deemed to be negative or undesirable. While the objective probability of something occurring and its expected consequence can be calculated in some cases, people’s perceptions of these two factors may deviate from the objective estimate (Mousavi & Gigerenzer, 2014; Tversky & Kahneman, 1979). Perceived risk may thus be considered a subjective evaluation of the probability and the consequence of an occurrence (Dowling & Staelin, 1994).

The psychological aspects of risk perception as a predictor of behavior can be complex and multifaceted. Several studies have examined the role of perceived risk on health-related compliance (Cori et al., 2020). This research suggests that when people perceive the risk of a negative health outcome as high, they may be more likely to take precautionary measures, whereas when the risk is perceived as low, compliance with those measures tends to be lower. Perceived risk is an important predictor of various precautionary health-behaviors, such as wearing facemasks and taking vaccines (Brewer et al., 2007; Schwarzer, 2001; Van der Pligt, 1998). The core assumption in this research has been that people are more motivated to adhere to infection control measures if they see themselves to be at risk.

In a pandemic setting, the personal risk may correspond to how people assess the likelihood of being infected and the severity of contracting the disease. Research on the H1N1 pandemic in 2009 indicated that seeing the risk of infection as high was associated with taking precautionary measures against infection (Bults et al., 2011; Walter et al., 2012). More recent research on the COVID-19 pandemic has found a similar relationship between perceived risk and adherence to infection control measures (Webster et al., 2020). For instance, protective behavior such as hand washing and social distancing was associated with the perceived probability of being infected with COVID-19 during the first week of the outbreak in the US (Wise et al., 2020). Seeing COVID-19 as a threat predicted protective measures during the UK lockdown (Brown, Coventry & Pepper, 2021). Similar patterns have been shown across several countries and during different stages of the pandemic (*e.g.*, De Bruin & Bennett, 2020, in the US; Ning et al., 2020, in China; Rattay et al., 2021, in Germany). COVID-19 risk perceptions was associated with the greater adoption of protective behavior across countries in Europe, America and Asia (Dryhurst et al., 2020).

However, the motivation to comply with infection control measures may only partly be driven by people’s self-interest in safeguarding their own health and partly driven by the desire to help or protect others (Aydinli et al., 2014). The Dryhurst et al. (2020) study cited above showed that an individualistic worldview, prosocial intentions and personal experience with the virus were predictors of risk perception. Studies on compliance to COVID-19 control measures has shown that prosocial concerns (being concerned about others’ health), and a sense of social responsibility predict engagement with infection control measures (Banker & Park, 2020; Böhm & Betsch, 2022; Zaki, 2020). This dual motivation to protect oneself and to protect others underscores how risk assessments also need to account for social considerations when shaping compliance to infection control measures. It should be noted that most of these studies used a cross-sectional design.

Cross-sectional data on perceived risk and compliance present limitations in establishing causal relationships and discerning the directional nature of the association between the variables. In one of few longitudinal studies on this, Schneider et al. (2021) measured risk perception of COVID-19 and health protective behaviors over a 10-month period in the UK through five nationally balanced cross-sectional surveys. The results revealed a consistent positive correlation between risk perception and adoption of protective health behaviors, where the strength of the relationship varied over time. They also found psychological factors to be more predictive of risk perception than the objective measures such as the rate of new cases (Schneider et al., 2021). Lages et al. (2021) investigated this relationship during outbreaks of avian influenza, seasonal influenza and the common cold in Germany. They found that risk perceptions matched the relative level of risk associated with these diseases, and that during the period when influenza and the common cold were more prevalent, participants felt more at risk for these diseases compared to off-season times. However, this increase in perceived risk was more noticeable when considering others rather than themselves (Lages et al., 2021). This fits with a fallacy known as optimistic bias, whereby people systematically ascribe higher risks to others than they do to themselves across situations (Sharot, 2011; Van der Pligt, 1998). While the authors found a relationship within each data collection round, panel data is needed to analyze for temporal relationships between risk perception and compliance during a pandemic event.

#### Compliance with infection control measures

Compliance with infection control measures is a key component of preventing the spread of infectious diseases. Measures such as hand hygiene, social distancing, and use of protective equipment, are essential for protecting society at large as well as individuals who are at particular risk. However, it may be challenging for individuals to comply with numerous measures to mitigate infection spread as it often requires individuals to change their behavior in inconvenient ways and adopt new habits. In the context of COVID-19, compliance with infection control measures and engagement in preventive behavior are intricately intertwined, often overlapping considerably. Both concepts entail actions aimed at reducing the spread of the virus and minimizing individual and collective risk. Compliance with measures such as wearing masks, practicing hand hygiene, and maintaining social distancing constitutes a proactive approach to preventing transmission, aligning closely with behaviors typically associated with preventive action. Consequently, in much of the research on COVID-19, these terms are frequently used interchangeably to represent the collective efforts individuals undertake to mitigate the impact of the pandemic (Brouard, Vasilopoulos & Becher, 2020; Burton et al., 2021; Clark et al., 2020; Harper et al., 2021).

According to the health belief model (Janz & Becker, 1984), in order for health-related behavior change to happen, there must be a combination of health concern, perceived threat, perceived benefits of change, and an absence of perceived barriers or cost. The model suggests that individuals are more likely to engage in health protective behaviors when they believe they are at risk, when they perceive the health problem to be serious, and when they believe that acting will reduce the risk and it is feasible to do so. In a pandemic setting this may correspond to being more likely to comply with infection control measures if they have a high level of motivation to protect their health, perceive a considerable level of threat from the virus, and believe that following recommended measures will effectively reduce the threat. A systematic review found that using the health belief model was effective as a theoretical basis for designing interventions that increased adherence with health recommendations (Jones, Smith & Llewellyn, 2014), and COVID-19 vaccination intention has been predicted by the model constructs (Wong et al., 2020).

Over the course of a pandemic, factors that affect compliance with infection control measures may shift. One such factor is motivation, which can be influenced by personal responsibility, belief in the effectiveness of measures, and positive reinforcement. Those who understand the importance of measures, such as wearing facemasks, and receive positive feedback may be more motivated to comply. Research on the H1N1 influenza showed that while knowledge about the disease increased, both perceived risk and intention to comply with control measures decreased over the first four months of the virus spread in the Netherlands (Bults et al., 2011). A study of self-reported compliance with COVID-19 social distancing measures over three months in the US, found that compliance was influenced by intrinsic motivation, capacity to comply, impulse control, social norms, and perceived duty to obey rules (Reinders Folmer et al., 2021). They also found that compliance declined over the course of the three months and that the decline was associated with people’s threat perceptions, knowledge, and perceived social norms.

As the pandemic progressed, perceptions of risks related to COVID-19 may have changed for some individuals. Some may have become complacent about the situation, while others may have become more fearful as the number of cases rose and new variants of the virus emerged. Through the first year of the pandemic, new information and guidelines were periodically released, and this may have affected people’s confidence in and motivation for complying with the infection control measures. On the other hand, people may have gained confidence in the control measures as it may have prevented them from getting infected with COVID-19, and they may have ascribed this to the effectiveness of precautionary behavior. As a consequence, compliance with infection control measures may contribute to perceiving less risk of getting infected with COVID-19. Understanding how perceptions of risk and compliance change over time may be crucial for developing interventions that will encourage compliance with infection control measures.

### Knowledge gap

The majority of the studies on perceived risk and compliance during the first COVID-19 pandemic is based on cross-sectional surveys on convenience samples (Dryhurst et al., 2020), and a systematic review found that over 95% of studies about risk awareness in regard to containing COVID-19 used a cross-sectional design (Cipolletta, Andreghetti & Mioni, 2022). While cross-sectional studies are useful, it may be challenging to represent the motivations involved in a dynamic phenomenon such as a pandemic into an assessment on a single time-point. Particularly in a rapidly changing situation such as a global pandemic, it is important to investigate the stability of relationships over time, both from a theoretical and practical perspective. It has previously been argued that longitudinal studies on risk perception are necessary as cross-sectional studies can lead to wrong conclusion about the relationship between perceived risk and mitigation behaviors (Loewenstein & Mather, 1990; Siegrist, 2013). Also, the sampling bias involved in using convenience samples may create an inaccurate impression of how people perceive pandemic risks and their level of compliance that may not hold for the population at large. The combination of perceived risk and compliance with health protective measures are rarely studied longitudinally, and the reverse relationship, with compliance as the predictor and risk as the outcome, is less documented still in the literature. While we should expect perceived risk to predict compliance, less is known about how past engagement with protective behavior may impact how people perceive of the risk scenario. The current study fills this knowledge gap by investigating the relationship between perceived risk and compliance using longitudinal data from a representative sample.

### Current study

The current study was conducted as a part of the PANDRISK research project that aimed to measure, track and predict the effect of perceived risk on compliance during the COVID-19 pandemic in Norway (see website: https://www.uib.no/en/pandrisk). The current study used data from four nationally representative survey data collections collected between March 2020 and November 2020. Similar to Schneider et al. (2021), we measured the perceived risk of infection and compliance to infection control measures over the first year of the pandemic in Norway, but unlike the Schneider et al. (2021) we used a panel design that allows for temporal analyses between the variables. Longitudinal analysis allowed us to look for causal predominance between the variables and determine if the relationship changes over time. The current study used a registered report approach (Grand et al., 2018).

#### Timeline for COVID-19 spread and management of infection control measures in Norway

Norway adopted a strategy of controlling the spread of COVID-19 through measures such as restrictions on public gatherings, border closures, quarantine requirements for travelers, and widespread testing and tracing. The Norwegian health authorities also prioritized protecting vulnerable groups and avoiding a complete lockdown, instead opting for targeted measures and temporary closures of specific sectors as needed (NOU, 2022). On March 12th, 2020, Norway closed its borders to travelers from abroad, except for those with a residence or work permit, and the Norwegian government recommended that people work from home if possible and that schools and universities switch to remote learning. Norwegians were advised to adopt a number of personal hygiene measures such as handwashing, avoiding touching public surfaces, and keeping physical distance from others. These efforts helped Norway maintain a relatively low number of cases and deaths compared to many other countries in the initial phase of the pandemic (Ursin, Skjesol & Tritter, 2020). In addition to these measures, Norwegian authorities rolled out widespread testing and contact tracing efforts, and temporarily closed specific sectors, such as bars and restaurants, when outbreaks occurred.

Norway lifted many of its COVID-19 restrictions over time as the spread of the virus was brought under control. Starting in June 2020, the country lifted restrictions on social gatherings and allowed for some reopening of bars and restaurants, with restrictions on capacity and distancing measures in place (NOU, 2022). Throughout the rest of the year and into 2021, further easing of restrictions was carried out in a gradual manner, with close monitoring of the situation and adjustments made as needed.

#### Research questions and hypotheses

Based on previous research, there is reason to expect a positive association between “Perceived risk” and “Compliance”. We first tested whether there was a positive association between perceived risk and compliance with infection control measures within each measurement point (H1). Note that the association between ‘Perceived risk’ at T1 and ‘Compliance’ at T1 will not be tested as a confirmatory hypothesis in the current model, as it was previously examined in Sætrevik & Bjørkheim (2022) using somewhat different indices of risk (including affective worry) and compliance (including efficacy beliefs, trust in government, and prosocial motivation). . Next, we examined the temporal relationships between perceived risk and compliance with infection control measures to test whether perceived risk at the immediate prior measurement period, is positively associated with compliance at the subsequent measurement period (H2). This was tested for all four data collection rounds. The same analyses were run to test whether compliance at the immediate prior measurement point was associated (non-directionally) with perceived risk in the subsequent measurement point (H3).

H1: “Perceived risk” has a positive association with “Compliance” at each data collection.

(a) “Perceived risk” at T2 will have a positive association with “Compliance” at T2.
(b) “Perceived risk” at T3 will have a positive association with “Compliance” at T3.
(c) “Perceived risk” at T4 will have a positive association with “Compliance” at T4.

H2: “Perceived risk” at a prior measurement point will predict “Compliance” at the subsequent measurement point.

(a) “Perceived risk” at T1 will have a positive association with “Compliance” at T2.
(b) “Perceived risk” at T2 will have a positive association with “Compliance” at T3.
(c) “Perceived risk” at T3 will have a positive association with “Compliance” at T4.

H3: “Compliance” at a prior measurement point will predict “Perceived risk” at the subsequent point.

(a) “Compliance” at T1 will be associated with “Perceived risk” at T2.
(b) “Compliance” at T2 will be associated with “Perceived risk” at T3.
(c) “Compliance” at T3 will be associated with “Perceived risk” at T4.

## Methods

### Participants

The data for this study are from the “Norwegian Citizen Panel” (https://www.uib.no/en/citizen), which is a continuously running online panel survey of Norwegians’ opinions on social matters. Individuals are randomly drawn from the Norwegian Tax Registry and invited to participate in the panel. The survey has been fielded two to three times a year since 2013. In 2020, two additional survey rounds were fielded with questions about the COVID-19 pandemic. The panel aims to be representative for adult (above the age of 18) Norwegians, with minor deviations from perfect representativity in terms of age, education level and geographical regions. Across survey rounds, the deviations from a perfectly representative sample of the Norwegian population remain stable and relatively small (see methodology reports: https://osf.io/drzck/). People over the age of 60 are overrepresented by a margin of 16%, whereas those under 29 years old are underrepresented by 13%. The overrepresentation of individuals with a university or college degree is modest at 29%, while those with upper secondary education are underrepresented by around 10%, as well as those with elementary education by 19%. Geographically, the sample displays a slight (3–5%) overrepresentation of individuals from urban areas in Oslo, Akershus and Western Norway. These stable deviations from representativity suggest that the sample remains reasonably representative of the Norwegian population across survey rounds but is slightly older and more educated.

An anonymized version of the dataset devoid of personal identification codes, along with all analysis code has been made available and disseminated *via* the project’s Open Science Framework (OSF) page (https://osf.io/5k7qw/). The OSF repository includes separate components for data, code, and supplementary materials to facilitate navigation. Various precautions aimed at bolstering privacy are inherent in the dataset as all background demographic attributes are measured at a group level. A weighting variable for demographic deviations is provided in the dataset (https://osf.io/5k7qw/).

Data for this study were collected in four rounds, in March (T1, *n* = 4,083), June (T2, *n* = 2,820), August–September (T3, *n* = 5,541) and November (T4, *n* = 2,533) in 2020. We assume that most (90%) of the participants remain from one round to the next based on prior tendencies in the Norwegian Citizen Panel data. However, for rounds T1 and T3, the survey was fielded to a larger share of the Norwegian Citizen Panel, resulting in a sample size of 4,083 and 5,541 for these rounds respectively. The model was run on the participants who answered all the items in all rounds (complete cases). We expected that this approach would yield a panel of n ∼2,000. We compared the results of the complete case sample with different ways of handling missing data (both listwise deletion and pairwise deletion). Following Mulder (2023), a sample of 1,800 is sufficient to reliably detect “small” cross lagged effects of .10, at a power of .80 across four measurement rounds, even with a very high degree of between-unit variance.

### Data collection

The University of Bergen serves as the governing body for the Norwegian Citizen Panel, while the company Ideas2evidence is responsible for recruiting participants, designing the survey, and documenting the data collection process. Prior to data collection, the Norwegian Citizen Panel obtained written informed consent from all panel members, and all ethical considerations regarding data collection and storage were approved by the Norwegian Centre for Research Data (reference number: 118868). Participants were invited to the data collection by email, with a reminder being sent out a week later to those who had not opened or completed the survey. A second reminder by email is typically sent out one week after the first reminder, and this is followed by a third reminder by sent out by SMS a few days later (see methodology reports for detailed description: https://osf.io/drzck/). Most participants (75%) typically respond within a week of receiving the survey. The panel data allows for analysis between data collection rounds. We assume an attrition rate from one round to the next of less than 5%, as this is the average wave-to-wave retention rate in the panel. No observations in the dataset were excluded from the analysis. The Citizen Panel removes participants who have not responded to any of the last three survey rounds from the final datasets. These respondents were not part of our analyses. The authors involved in this study did not have control over the data collection process (preempting the possibility of optional stopping).

### Materials and variables

Two variables were part of this analysis. The first variable, “Perceived risk” was measured with the average of four items asking about the risk of infection for self and others, risk of becoming seriously ill, and risk for changes to everyday life. The second variable, “Compliance” was measured with one item asking about the overall intention to follow the infection control measures. Because compliance was measured with a single item repeated across waves, it should not be considered a latent construct with high internal consistency. Instead, it provides a direct repeated assessment of participants’ self-reported adherence to infection control measures at each time point. See Table 1 below for the phrasing of the items constituting the variables. The items were measured using a Likert-type scale with five response options for each statement. The perceived risk items asked participants to rate the level of risk and assigned numerical values between very low (1), somewhat low (2), medium (3), somewhat high (4), and very high (5). The compliance item was measured with five response options and assigned numerical values ranging from completely disagree (1), disagree (2), neither agree nor disagree (3), agree (4), and completely agree (5). We tested the hypotheses listed above by indexing the four perceived risk items and used a multiverse analysis to assess the robustness of the findings.

**Table 1:** List of items.

Variable	Item text (translated to English)
Perceived risk	How high or low do you think the risk is that you will be infected by the coronavirus?
Perceived risk	How big do you consider the risk that an average adult will be infected by the coronavirus?
Perceived risk	How big do you consider the risk that you will become seriously ill from the coronavirus?
Perceived risk	How big do you consider the risk that your everyday life will change a lot due to the coronavirus?
Compliance	I do my best to follow the various advice from health authorities to limit the risk of infection (often washing hands, avoiding travel and situations with other people, keeping my distance and avoiding touching things)

**Figure 1:**
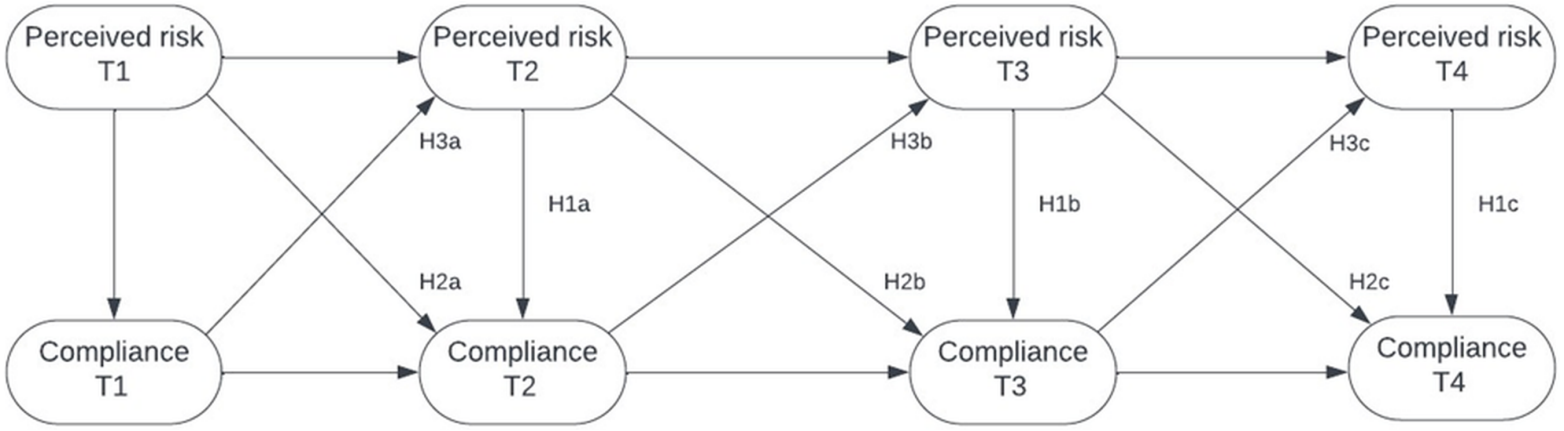
Random intercept cross lagged panel model with hypotheses shown. Simplified representation of the hypotheses within the random intercept cross-lagged panel model (RI-CLPM) of perceived risk and compliance 4 across the four measurement rounds.

### Analysis plan

We ran a random intercept cross-lagged panel model (RI-CLPM, Mulder & Hamaker, 2021) on the data to test associations between perceived risk and compliance for the measurement points 1–4 (see Fig. 1). We also performed a multiverse analysis by testing the RI-CLPM model with different ways of combining the perceived risk items into an index. This left us with 15 possible combinations of the “perceived risk” variable (excluding the option where none of the items are counted towards the index), and enabled us to compare how robust the findings were to a particular operationalization of “Perceived risk”.

## Results

### Descriptive statistics

Upon receiving the in-principle acceptance (see Stage 1 URL: https://osf.io/nmxb8/), we combined the measurements of perceived risk and compliance to infection control measures from four time-points during the first year of the pandemic in Norway. In accordance with the preregistered analysis plan, we restricted our analyses to participants with complete data at all time points (see dataset here: https://osf.io/m8bgu/). This resulted in a final sample of *N* = 2,190 participants which was slightly above the originally estimated sample size (*n* ≈ 2,000) at the time of stage 1 registration. Overall, participants reported moderate levels of perceived risk at each wave, with the highest mean observed at T1 (*M* = 3.02, *SD* = 0.71) and the lowest mean at T2 (*M* = 2.13, *SD* = 0.66). Across the following measurement points, perceived risk remained relatively moderate (T3: *M* = 2.26, *SD* = 0.66; T4: *M* = 2.65, *SD* = 0.68). Participants reported consistently high intentions to comply across the study period. The highest mean was observed at T1 (*M* = 4.69, *SD* = 0.67) and the lowest mean at T4 (*M* = 4.34, *SD* = 0.94). Compliance remained in the high range at T2 (*M* = 4.40, *SD* = 0.82) and T3 (*M* = 4.42, *SD* = 0.76). It is noteworthy that compliance scores were consistently high across all waves, with means above 4 on a 5-point scale. This indicates a potential ceiling effect that may have constrained variability in compliance responses. Internal consistency was good for the perceived risk index across the four items (Cronbach’s *α* = .81 at T1). Compliance was measured with a single item at each wave (see Table S3 in appendix for correlations between the variables).

### Model fit

A RI-CLPM (Mulder & Hamaker, 2021) was specified to examine the associations between perceived risk and compliance across four time points (T1, T2, T3, T4). All analyses were conducted in RStudio using the lavaan package (see analysis script here: https://osf.io/c7ygx). The RI-CLPM showed good fit to the data, *χ*2(9) = 43.565, *p* < .001. Additional fit indices (*CFI* > .95, *RMSEA* < .06, and *SRMR* < .08) supported the adequacy of the model (Hu & Bentler, 1999). Following standard RI-CLPM conventions, each construct (*i.e.,* perceived risk and compliance) was modeled to have a latent random intercept factor. This factor represents stable, trait-like individual differences across measurement rounds. The covariance between the random intercepts of perceived risk and compliance was not statistically significant (*σ* = 0.012, *SE* = 0.007, *p* = .068). Thus, once stable between-person differences were accounted for, there was no evidence of a trait-level relationship between perceived risk and compliance. In accordance with the analysis plan, we ran the model using alternative methods for handling missing data (listwise and pairwise deletion). These additional analyses did not change whether or not the hypotheses were supported when compared with the “complete cases” approach reported below.

### Confirmatory hypothesis testing

#### Hypothesis 1: “Perceived risk” has a positive association with “Compliance”  within each data collection

The number of participants was constant across all confirmatory tests (*N* = 2,190).

H1a: We hypothesized that perceived risk at T2 would have a positive association with compliance at T2. Contrary to this expectation, the association between perceived risk and compliance at T2 was negative and did not reach the predetermined level of significance, (*β* =  − 0.01, SE = .01, z = −1.69, *p* = .090; standardized *β* = −.05). Consequently, we did not find support for H1a.

H1b: The same prediction was made for T3. The result at T3 was again nonsignificant, and the coefficient was effectively null (*β* = .00, SE = .00, *z* = 0.97, *p* = .329; standardized *β* = .02). Consequently, we did not find support for H1b.

H1c: We made the same prediction for T4. Contrary to our expectation, the association was statistically significant but negative (*β* =  − 0.03, SE = .01, z = −3.54, *p* < .001; standardized *β* = −.08). This suggests that at T4, participants with elevated perceived risk reported lower compliance relative to their own typical levels. This is contrary to the predicted positive association and thus H1c was also not supported.

Taken together, these findings did not show support for the anticipated positive association between “Perceived risk” and “Compliance” within each data collection round as predicted under H1. Rather, the only significant association was in the opposite (negative) direction.

#### Hypothesis 2: “Perceived risk” at one measurement point will predict “Compliance” at the subsequent measurement point

H2a: We hypothesized that higher perceived risk at T1 would predict higher compliance at T2. However, the cross-lagged parameter (*β* =  − 0.05, *SE* = .04, *z* =  − 1.30, *p* = .193; standardized *β* = −.03) was nonsignificant, negative, and very small, offering no support for H2a.

H2b: Similarly, we hypothesized that higher perceived risk at T2 would predict higher compliance at T3. This path was effectively null (*β* = 0.00, *SE* = .04, *z* = 0.04, *p* = .965; standardized *β* = .00), offering no support of a positive influence from perceived risk to compliance at the subsequent time point.

H2c: Finally, we hypothesized that higher perceived risk at T3 would predict higher compliance at T4. The estimate (*β* =  − 0.08, *SE* = .04, *z* =  − 1.81, *p* = .070; standardized *β* = −.04) was nonsignificant and trended in a negative direction, contrary to H2c’s positive prediction. Consequently, we did not find support for H2c.

Hence, “Perceived risk” did not exhibit a statistically significant prospective association with “Compliance” in any of the rounds thus failing to support H2.

#### Hypothesis 3: “Compliance” at one measurement point will predict “Perceived risk” at the subsequent point

H3a: We hypothesized that compliance at T1 would predict perceived risk at T2. However, the cross-lagged path (*β* =  − 0.00, *SE* = .02, *z* =  − 0.11, *p* = .911; standardized *β* = −.00) was close to null, nonsignificant, and thus offering no support for H3a.

H3b: Similarly, we hypothesized that T2 compliance would predict perceived risk at T3. This path was again minimal (*β* =  − 0.01, *SE* = .01, *z* =  − 0.70, *p* = .479; standardized *β* = −.02), offering no support for an association between compliance and subsequent perceived risk.

H3c: Lastly, we investigated whether compliance at T3 would predict perceived risk at T4. This path was again minimal (*β* =  − 0.01, *SE* = .01, *z* =  − 0.85, *p* = .392; standardized *β* = −.02), thus offering no support for H3c.

Hence, “Compliance” did not exhibit a statistically significant prospective association with “Perceived Risk”, and thus failing to support H3 in any of the measurement rounds.

### Multiverse analysis

We conducted a multiverse analysis to examine whether the results were influenced by different ways of operationalizing the variable perceived risk (see analysis script here: https://osf.io/r3e4h). The four items originally used to measure perceived risk in the main model reported above, were combined in 15 different ways to create alternative perceived risk measures, each of which was then tested using the same random-intercept cross-lagged panel model (see Table 2 in the appendix). Across these 15 versions, we observed no new statistically significant associations, and the coefficients remained very small and in the same direction for most versions (see Table 3 and 4 in the appendix).

The cross-sectional associations (H1a, H1b) yielded consistent null findings at T2 and T3, but contrary to our hypotheses H1c we observed a negative association at T4. This indicates that participants who perceived higher risk reported lower compliance at that time point. The multiverse analysis replicated this statistically significant path in ten of the 15 versions at *p* < .01. Additionally, all the 15 versions produced a small negative coefficient (*β* =  − .06 to −.01) for this relationship and 11 of the versions had a beta coefficient at or above the .03 threshold (see h1c in Table S3 in the appendix). Closer inspection revealed that the negative coefficient was statistically significant when items assessing the likelihood of infection (for oneself or the average adult) were included, either individually or combined in indices. In contrast, items addressing the risk of serious illness or substantial changes to daily life did not predict compliance at this point, whether considered alone or combined in indices (see for instance version four, five and 11 in Table S4 in the appendix). Thus, the negative association appears to be driven by how individuals evaluated the likelihood of contracting COVID-19 for themselves and for others.

Across all 15 alternative perceived risk measures, none yielded statistically significant cross-lagged paths between perceived risk and subsequent compliance (H2a–c), nor between compliance and subsequent perceived risk (H3a–c). In sum, the multiverse analysis confirms that the null findings are robust to different operationalizations of perceived risk, indicating that the overall null results do not hinge on specific measurement choices but rather reflect a consistent pattern in the data.

## Discussion

This study examined whether perceived risk of COVID-19 was associated with compliance with infection control measures during the first year of the pandemic in Norway. We investigated this relationship in a representative panel of Norwegian adults using four data collection waves between March and November 2020. Classical frameworks like the health belief model (Janz & Becker, 1984) and the protection motivation theory (Floyd, Prentice-Dunn & Rogers, 2000; Rogers, 1975) propose that individuals who perceive higher health risks are more inclined to adopt preventive behaviors. Empirical studies support this theoretical link by demonstrating a positive association between risk perception and compliance with health protective measures (*e.g.*, Brewer et al., 2007; Cipolletta, Andreghetti & Mioni, 2022; Dryhurst et al., 2020; Ferrer & Klein, 2015; Webster et al., 2020). Drawing on these theoretical and empirical foundations, we hypothesized that higher perceived risk of COVID-19 would predict higher compliance with infection control measures during the pandemic. Contrary to expectations, we found no consistent evidence of a positive association between perceived risk and compliance at any of the measurement points (H1), nor did perceived risk predict subsequent compliance at a later time point (H2). Also, compliance did not predict subsequent risk perception (H3).

A critical contribution of this study was the examination of how perceived risk relates to compliance across different phases of the pandemic. Specifically, data were collected at four distinct time points: the initial outbreak phase, a dormant phase with reduced infection rates, a resurgence phase marked by renewed spread, and a later stage characterized by high infection rates but potentially greater public adaptation to pandemic conditions. This temporal differentiation is important, as risk perceptions and compliance motivation can shift in response to new information, changing government regulations, COVID-19 spread, and media coverage (Reinders Folmer et al., 2021; Schneider et al., 2021). In the early months of an infectious outbreak, perceived risk and adoption of countermeasures typically align (Bults et al., 2011; Wise et al., 2020), possibly due to heightened alarm and awareness. This pattern has also been documented during the initial phase of the COVID-19 pandemic in Norway (Sætrevik & Bjørkheim, 2022). However, while people in the current study saw the risk as higher at T1 than at any other measurement point, this did not translate into a positive association with T2 compliance. Over time, pandemic fatigue and shifting social norms may have weakened or even reversed the association between risk perception and compliance with control measures. Our observation of a negative association at T4 may reflect that some participants became fatigued or perhaps questioned the effectiveness of measures or felt overwhelmed by prolonged restrictions despite seeing the risk of infection as high. Conversely, participants with lower perceived risk at T4 might have maintained their compliance due to alternative motivations such as social responsibility or protecting vulnerable community members (Aydinli et al., 2014; Böhm & Betsch, 2022; Dryhurst et al., 2020).

These findings diverge from several cross-sectional studies conducted early in the COVID-19 pandemic (*e.g.*, Brown, Coventry & Pepper, 2021; Wise et al., 2020) and during previous health crises (Bults et al., 2011; Walter et al., 2012), where higher perceived risk typically correlated with greater preventive behaviors. They also differ from the pattern reported by Schneider et al. (2021), who observed consistent positive associations between risk perception and protective behavior over a 10-month period in the United Kingdom. A critical methodological difference may explain this discrepancy: Schneider et al. (2021) used repeated cross-sectional surveys, while our panel design specifically captured within-person changes, distinguishing these fluctuations from stable between-person differences. Cross-sectional or repeated cross-sectional analyses might reveal broad group-level associations, yet mask individual trajectories and person-specific stability in risk perception. Despite these methodological distinctions, our findings consistently demonstrate little to no correlation between perceived risk and compliance throughout the first year of the pandemic in Norway.

The present study also addressed a typically underexplored directional association from compliance to risk perception. It was hypothesized that compliance over time might influence perceived risk, based on the assumption that engaging in protective behaviors would help individuals feel safer and lower their perceived threat of infection (Brown, Coventry & Pepper, 2021). Contrary to this expectation, our findings showed that prior compliance did not significantly influence subsequent risk perception. The absence of significant cross-lagged paths after accounting for stable between-person differences indicates that individual changes in compliance might have minimal impact on future risk perceptions, suggesting that perceptions of risk might instead be shaped predominantly by immediate contextual factors rather than past behavior.

The robustness of these findings was further assessed through a multiverse analysis, which examined 15 alternative operationalizations of perceived risk to ensure comprehensive coverage of potential measurement variations. Each alternative measure was subjected to the same analytical procedures to confirm the stability and consistency of the null findings. There were no significant new associations in any of the 15 versions, and the direction and magnitude of the coefficients changed only minimally. The consistent null results across these analyses indicate that our findings from the confirmatory model were not contingent upon specific measurement choices. However, one notable pattern emerged at T4 (H1c), where perceived risk was unexpectedly negatively related to compliance. This finding from the confirmatory model was replicated in most of the alternative versions, showing that individuals who reported higher risk perceptions at T4 tended to report lower compliance at the same point. Although this result does not align with the common assumption that greater risk perceptions lead to more protective behavior, it appeared in ten of the 15 alternative multiverses. Upon closer examination, the negative association appeared in versions that relied on the items measuring likelihood of infection for the individual and the average adult, while items related to serious illness or major daily disruptions did not predict compliance (see Table S3 and S4 in appendix). This suggests that the perceived risk of infection may shape behavior in ways that differ from perceived risk of severe illness and changes to daily life. It is also notable that we do not observe the same pattern at T2 or T3, indicating that something distinctive about the conditions at T4 may have contributed to the negative association. Indeed, T4 coincided with a period of substantially higher COVID-19 spread than at any previous point. One possibility is that people felt an inevitability about becoming infected or experienced increased fatigue by that time, potentially leading to a negative link between infection risk assessments and compliance with protective measures.

### Robustness checks and theoretical considerations

Overall, the unexpected null findings highlight the complexity surrounding perceived risk and compliance within an extended health crisis. The interplay between these constructs likely involves multiple situational factors (*e.g.*, official regulations, news media emphasis, behavioral norms) and personal factors (*e.g.*, altruistic motivations, personality traits, interpersonal trust, coping capacity, or evolving experiences with the virus). For instance, prosocial motivations, rather than personal fear, can be a critical driver of compliance with infection control measures (Banker & Park, 2020; Zaki, 2020). Therefore, individuals may maintain high levels of compliance out of empathy or communal concern, even if their perceived risk to themselves is minimal. This dynamic could possibility conceal associations between perceived risk and compliance. However, the second version of our multiverse analysis tested whether perceiving risk for others was associated with compliance, and we found no positive relationship in this model. This suggests that participants who saw a higher risk for others did not necessarily intend to comply more, which challenges the idea that prosocial motivation alone conceals the link between risk perception and compliance in our data. On the other hand, it remains possible that concern for close family or specific social groups (factors we did not directly measure) plays a more important role. Lastly, our findings challenge the predictive power of classical frameworks such as the health belief model and protection motivation theory in ongoing, evolving pandemic contexts, suggesting these models may better apply to acute or static health threats. Although theoretical models such as the health belief model and protection motivation theory are typically framed in causal terms (*i.e.,* that higher perceived risk leads to stronger compliance), our observational panel design does not allow us to make definitive causal claims. As Rohrer (2018) emphasized, associations between psychological variables can reflect a range of possible causal structures, including confounding and collider effects. In our context, risk perception could plausibly influence compliance (*e.g.*, perceiving high infection risk motivates adherence), while compliance might also influence subsequent perceptions of risk (*e.g.*, protective behavior provides reassurance and lowers perceived threat). At the same time, both variables may be shaped by unmeasured factors such as trust in authorities, prosocial motivations, personality traits, or media exposure. The current study therefore informs the temporal association between risk perception and compliance, but should not be taken as evidence of causal direction. Moreover, our results echo calls (Loewenstein & Mather, 1990; Siegrist, 2013) for more longitudinal designs, demonstrating that cross-sectional evidence of correlation from an initial outbreak does not always generalize to within-person, time-sequenced relationships. Overall, the patterns we observe imply that perceived risk alone may not explain or motivate people to sustain compliance over long durations in pandemic settings, at least in the Norwegian context.

### Limitations and future research

Several limitations should be considered when interpreting the findings of this study, as they may influence the validity and generalizability of the results. One limitation was that compliance with infection control measures was assessed using self-reported intentions rather than measures of actual behavior. Consequently, we do not know the extent to which participants actually complied, only their reported intention to do so during the pandemic’s first year. Self-reporting can introduce social desirability bias (Edwards, 1957; Krumpal, 2013), potentially affecting participants responses. Indeed, our data indicated a strong skew towards high reported compliance, which might reflect social norms that encourage positive responses or stigma associated with reporting non-compliance. Such social desirability bias could have created a ceiling effect, limiting our ability to detect any relationship between perceived risk and compliance and possibly explaining why we did not observe this relationship. This interpretation aligns with the Scandinavian high-trust context, where widespread willingness to comply with public health guidelines may have reduced between-person differences in compliance responses. Previous work has shown that motivational factors such as prosocial orientation and contextual factors such as trust in authorities were positively related to compliance in Norway (Sætrevik & Bjørkheim, 2022). This suggest that these may be more important drivers of compliance than risk perception alone.

A further limitation is that compliance was assessed with a single self-report item. This approach was chosen to ensure comparability across waves. However, it may not have been fine-grained enough to capture variation across different types of compliance behaviors (*e.g.*, distancing, hygiene, or mask use). Moreover, single-item self-report does not allow for strong internal consistency or stable factor loadings across time. Thus, compliance should be interpreted as a time-specific measure of self-reported adherence, rather than as a latent trait. Future studies could strengthen measurement by including multi-item compliance scales or behavioral indicators that provide a more reliable and nuanced assessment of the construct.

Data were collected at four discrete time periods during the pandemic, which may not adequately capture day-to-day fluctuations in compliance and perceived risk. This methodological approach might obscure short-term, context-driven changes which may be crucial for understanding individuals’ responses to evolving public health conditions. Behavior and perceptions can shift rapidly due to personal circumstances (*e.g.*, changes in employment or contracting COVID-19) or external factors (*e.g.*, emerging virus variants, updated policies, public health communications). Because these shifts were not captured as they occurred in real time, our findings may not reflect the full range of variability in behavior and risk perceptions. Therefore, we cannot confidently determine whether changes in participant behavior or perceptions were driven by psychological processes or by evolving external circumstances. Future research could benefit from more frequent measurements, such as daily diaries or experience sampling, to better understand how specific events influence the relationship between compliance and perceived risk.

This study was conducted in a Scandinavian context characterized by high interpersonal trust and high trust in authorities, conditions often associated with greater adherence to public health guidelines. Future studies should include cross-cultural comparisons to examine whether the observed relationship between perceived risk and compliance generalizes to other settings or differs in regions with varying public health strategies, institutional trust, and cultural norms.

A further limitation is that our study was not designed to distinguish between causal pathways. Although we examined temporal associations, we did not include potential confounders (*e.g.*, trust in institutions, prosocial orientation, political ideology) or colliders (*e.g.*, personal experiences with infection, government policy changes) that could shape both perceived risk and compliance. As Rohrer (2018) noted careful causal inference requires explicit modeling of such factors. Future studies could extend this work by incorporating additional variables to test whether the observed associations reflect direct causal effects, common causes, or other structural relations. Such an approach would allow stronger conclusions about whether and when perceived risk causes compliance, or vice versa.

## Conclusion

The current study from the first year of the pandemic in Norway did not demonstrate the anticipated positive associations between perceived risk and compliance with infection control measures. Instead, the results suggest that risk perceptions may not be a decisive driver of whether people comply with infection control measures. This finding runs counter to predictions derived from classical frameworks in health psychology, the majority of research on risk perception and protective behavior, and most comparable studies on compliance to control measures during the COVID-19 pandemic. The present results could indicate that the relationship between these constructs is less reliable than previously assumed, that other factors could play a more significant role in certain contexts, and that perceived risk may have less influence over time as individuals grow more accustomed to conditions involving health risks. One implication may be that health authorities and policymakers should look beyond instilling or heightening perceptions of personal danger when designing and communicating public health strategies in Norway.

## Supplemental Information

10.7717/peerj.20554/supp-1 Supplemental Information 1 Dataset with all variables

10.7717/peerj.20554/supp-2 Supplemental Information 2 Code for descriptives and correlation table

10.7717/peerj.20554/supp-3 Supplemental Information 3 Code for multiverse analysis

10.7717/peerj.20554/supp-4 Supplemental Information 4 Code for confirmatory analysis

10.7717/peerj.20554/supp-5 Supplemental Information 5 Appendix including tables and figures referenced in the PCI RR stage 1 and Stage 2 manuscripts

## Appendix

**Researchers’ prior knowledge of data (As stated in PCI RR stage 1 and Stage 2 submissions, but moved to appendix upon editor request for the final manuscript)**

The data has already been collected at the time of analysis planning, and the researchers have had access to the data. Some of the response distributions of items used in the current study have already been examined and reported. Firstly, response distribution for perceived risk and compliance at T1 has been examined and reported (Sætrevik, 2021) and we have previously described the cross-sectional association between perceived risk and compliance at T1 using somewhat different indices of the two constructs (Sætrevik & Bjørkheim, 2022). In that study, compliance included items on efficacy beliefs, trust in government, and prosocial motivation, and the perceived risk index also included affective components such as worry about the pandemic. These differences mean that the earlier test should be regarded as a conceptual rather than a direct replication of the association studied here. Contrary to our hypothesis, we found that those who perceive a higher risk are actually less likely to comply with safety measures. When we specifically looked at the risk of getting infected, we found a very small negative effect on compliance. However, when we asked about the perceived risk of infection for the general population, the effect on compliance was small and positive. Overall, our findings only weakly support the idea that seeing the pandemic as a threat leads to following safety measures, and only when considering the risk to the general population, not to oneself (Sætrevik & Bjørkheim, 2022). This association was not considered among confirmatory hypotheses in the current study. We have also calculated arithmetic means of the items suggested for this study at T1-T4, to report descriptive changes during the pandemic.

Note that in prior examination of the data we have not tested the relationship between perceived risk and compliance at any time points after T1, or tested associations between averages of the variables across T1-T4, or tested any cross-lagged associations (*i.e.,* between time points). Despite being naïve to how the data are related to the current research questions, our access to the data is classified as a “level 1″submission in the PCI RR table for prior knowledge.

To combat the risk of bias associated with a “level 1” submission, a multiverse analysis was performed by running the model with all possible combinations (except the option where none of the items are included) of items constituting the “perceived risk” variable. This led to 15 different pathways of analysing the model (see script: https://osf.io/yvz87/). This approach showed whether the results were robust towards different operationalizations of the “perceived risk” measure. The model was also analysed with different ways of handling missing data by testing the model using either listwise or pairwise deletion to examine whether the results were robust towards selective attrition. In addition to the multiverse approach, a “blinded analyst” approach was adopted. The second author functioned as a blinded analyst, as he had not had access to the data and had not been part of the data curation process. In practice, the first author anonymized the data by renaming the variables “perceived risk” and “compliance” to either “tango” and “foxtrot” for each time-point before transferring the dataset to the blinded analyst. The analyst could thus not know if the hypotheses were supported or not when reporting the analysis.

## Additional Information and Declarations

### Data Availability

The following information was supplied regarding data availability: The code (analysis script) and data are available at OSF: Bjørkheim, Sebastian, and Bjørn Sætrevik. 2023. “Relationship Between Perceived Risk and Compliance.” OSF. October 23. osf.io/5k7qw.
