# Supplementary material for: Relationship between perceived risk and compliance with infection control measures during the first year of a pandemic
Source: PeerJ. 2026 Feb 19;14:e20554. doi: 10.7717/peerj.20554 (PMC12925416; doi:10.7717/peerj.20554)
Supplement: Supplemental Information 5 [file peerj-14-20554-s005.docx]

**Table 2 (PCI Registered report study design template included in stage 1 manuscript)**

*Study design template*

| **Question** | **Hypothesis** | **Sampling** | **Analysis Plan** | **The rationale for deciding the sensitivity of the test for confirming or disconfirming the hypothesis** | **Interpretation given different outcomes** | **Theory that could be shown wrong by the outcomes** |
| --- | --- | --- | --- | --- | --- | --- |
| Is “perceived risk” positively associated with “compliance” within each data collection round? | H1 a-c (see description in section 1.3.2) | Nationally representative panel data, with four data collections. We only counted participants who answered all the items in all the rounds towards the analysis (complete cases approach). We expected a panel sample of n ~ 2000. We compared the results of this analysis to that of listwise deletion and pairwise deletion. | We ran a random intercept cross-lagged panel analysis on the data to test if there was an association between perceived risk and compliance within each of the measurement points 1-4.  We tested all the hypotheses against a p-value less than .01. In accordance with Orth et al (2022), we considered effect sizes of the RI-CLPM associations equal to or above 0.03 to be meaningful in the predicted direction.  We also performed a multiverse analysis by testing the RI-CLPM model with different ways of combining the perceived risk items into an index. This left us with 15 possible combinations of the “perceived risk” variable (we have excluded the option where none of the items are counted), and enabled us to compare how robust the findings are to a particular operationalization of “perceived risk”. | Since the mechanisms work on a population level, public health interventions that have small effects on compliance may nevertheless have considerable impact for a large number of individual’s health outcomes. Further, due to the potential for exponential growth in infection, small changes in compliance can have disproportionate effects on the population level. This lead us to accept small effect sizes as being relevant in this study. | Support for the H1 hypotheses would be taken to indicate that seeing a health crisis as a threat is associated with taking precautionary measures.  Lack of support for H1 hypotheses would indicate that seeing the risk as high at a given time during the health crisis did not motivate people to take precautions at that time.  The generalization of the interpretation of H1, H2 and H3 results may be limited to situations with similar pandemic severity, public knowledge, public health response and other cultural factors in Norway at the time of measurement. | If no relationships between perceived risk and compliance can be supported in the dataset, this could be discussed in light of the health belief model. |
| Does “Perceived risk” earlier in the pandemic predict “compliance” later in the pandemic? | H2 a-c (see description in section 1.3.2) |  | We ran a random intercept cross-lagged panel analysis on the data to test if there was an association between perceived risk at a measurement point and compliance at the immediate subsequent measurement point.  Cut-offs for significance and effect size of interest as above. |  | Support for the H2 hypotheses would be taken to indicate that seeing the risk as high at one point leads to taking more precautions later in the health crisis. This could be due to being concerned lead to establishing attitudes and to form habits for being cautious that are still present at a later time.  Lack of support for H2 hypotheses would indicate that perceived risk at an earlier stage does not impact behavior at a later stage. The inverse of H2 could also emerge, which would indicate that those who had been more concerned at an earlier stage were now fatigued or for other reasons less inclined to be cautious at a later stage. |  |
| Does “Compliance” earlier in the pandemic predict “Perceived risk” later in the pandemic? | H3 a-c (see description in section 1.3.2) |  | We ran a random intercept cross-lagged panel analysis on the data to test if there was an association between compliance at a measurement point and perceived risk at the immediate subsequent measurement point.  Cut-offs for significance and effect size of interest as above. |  | Support for the H3 hypotheses would be taken to indicate that being responsive to a health crisis at one point influences how the risk is viewed at a later time. A positive association could be due to a desire for current assessment to be consistent with previous behavior. A negative association could be due to disillusionment, after concluding that taking precautions about the risk in the past did not influence outcomes.  Lack of support for H3 hypotheses could be interpreted to indicate that taking precautions in earlier stages have no prolonged effect on how people see the risk from health crises at a later time. |  |

APPENDIX:

**Table 2:**

*Summary of Perceived Risk operationalizations for multiverse analysis*

Version Operationalization

Main model All items

Version 2 Includes only risk_infection_general

Version 3 Includes only risk_infection_self

Version 4 Includes only risk_sick_self

Version 5 Includes only risk_change_self

Version 6 Includes risk_infection_general and risk_infection_self

Version 7 Includes risk_infection_general and risk_sick_self

Version 8 Includes risk_infection_general and risk_change_self

Version 9 Includes risk_infection_self and risk_sick_self

Version 10 Includes risk_infection_self and risk_change_self

Version 11 Includes risk_sick_self and risk_change_self

Version 12 Includes risk_infection_general, risk_infection_self, and risk_sick_self

Version 13 Includes risk_infection_general, risk_infection_self, and risk_change_self

Version 14 Includes risk_infection_general, risk_sick_self, and risk_change_self

Version 15 Includes risk_infection_self, risk_sick_self, and risk_change_self

|  | Perceived risk  T1 | Perceived risk T2 | Perceived risk T3 | Perceived risk T4 | Compliance T1 | Compliance  T2 | Compliance  T3 | Compliance  T4 |
| --- | --- | --- | --- | --- | --- | --- | --- | --- |
| Perceived risk T1 | 1.00 |  |  |  |  |  |  |  |
| Perceived risk T2 | .47 | 1.00 |  |  |  |  |  |  |
| Perceived risk T3 | .41 | .53 | 1.00 |  |  |  |  |  |
| Perceived risk T4 | .45 | .50 | .61 | 1.00 |  |  |  |  |
| Compliance T1 | .07 | .03 | .03 | .03 | 1.00 |  |  |  |
| Compliance T2 | -.01 | -.02 | -.00 | -.01 | .19 | 1.00 |  |  |
| Compliance T3 | .04 | .03 | .05 | .02 | .14 | .28 | 1.00 |  |
| Compliance T4 | -.00 | -.01 | -.02 | -.06 | .16 | .24 | .22 | 1.00 |

**Table 3**

Correlation table of the eight measures used in the confirmatory analyses.

**Table 4**

*Beta coefficients for cross sectional (H1) and cross lagged (H2 and H3) relationships between Perceived risk and Compliance to infection control measures across 15 operationalizations of Perceived risk.*

| Hypothesis | Main Model | Version 2 | Version 3 | Version 4 | Version 5 | Version 6 | Version 7 | Version 8 | Version 9 | Version 10 | Version 11 | Version 12 | Version 13 | Version 14 | Version 15 |
| --- | --- | --- | --- | --- | --- | --- | --- | --- | --- | --- | --- | --- | --- | --- | --- |
| H1a | -.01 | -.02 | -.02 | -.00 | -.02 | -.02 | -.01 | -.02 | -.01 | -.02 | -.01 | -.01 | -.02 | -.01 | -.01 |
| H1b | .00 | .01 | .03 | .00 | -.00 | .02 | .01 | .01 | .00 | .00 | -.00 | .01 | .01 | .00 | .00 |
| H1c | -.03 | -.06 | -.04 | -.01 | -.02 | -.05 | -.03 | -.03 | -.03 | -.04 | -.01 | -.04 | -.04 | -.02 | -.03 |
| H2a | -.05 | -.04 | -.02 | .01 | -.01 | -.04 | -.01 | -.03 | -.03 | -.05 | -.01 | -.04 | -.05 | -.03 | -.04 |
| H2b | .00 | .01 | -.00 | -.01 | -.00 | .00 | .00 | -.00 | .00 | .00 | -.00 | .00 | .00 | -.00 | -.00 |
| H2c | -.08 | -.04 | -.05 | .01 | -.05 | -.07 | -.03 | -.09 | -.01 | -.08 | -.04 | -.04 | -.10 | -.08 | -.06 |
| H3a | -.00 | .05 | -.07 | .00 | -.00 | -.01 | -.02 | .00 | .03 | .02 | .01 | -.00 | -.01 | -.02 | .02 |
| H3b | -.01 | -.00 | .00 | .00 | -.05 | .00 | .00 | -.02 | -.00 | -.03 | -.02 | .00 | -.01 | -.01 | -.02 |
| H3c | -.01 | -.03 | -.01 | -.01 | .01 | -.02 | -.01 | -.00 | -.02 | -.01 | -.00 | -.02 | -.01 | -.00 | -.01 |

**Table 5**

P-values for cross sectional (H1) and cross lagged (H2 and H3) relationships between Perceived risk and Compliance to infection control measures across 15 operationalizations of Perceived risk.

| Hypothesis | Main Model | Version 2 | Version 3 | Version 4 | Version 5 | Version 6 | Version 7 | Version 8 | Version 9 | Version 10 | Version 11 | Version 12 | Version 13 | Version 14 | Version 15 |
| --- | --- | --- | --- | --- | --- | --- | --- | --- | --- | --- | --- | --- | --- | --- | --- |
| H1a | .090 | .165 | .136 | .914 | .201 | .123 | .246 | .088 | .262 | .090 | .301 | .146 | .075 | .130 | .127 |
| H1b | .329 | .435 | .011 | .913 | .685 | .050 | .081 | .237 | .578 | .952 | .803 | .116 | .253 | .338 | .940 |
| H1c | <.001 | <.001 | .001 | .195 | .178 | <.001 | .005 | .005 | .001 | .001 | .133 | <.001 | <.001 | .010 | .003 |
| H2a | .193 | .067 | .264 | .636 | .394 | .096 | .656 | .217 | .350 | .093 | .755 | .242 | .098 | .422 | .248 |
| H2b | .965 | .695 | .966 | .771 | .954 | .804 | .995 | .985 | .902 | .909 | .850 | .878 | .912 | .971 | .974 |
| H2c | .070 | .305 | .119 | .658 | .020 | .125 | .403 | .011 | .744 | .026 | .159 | .299 | .019 | .064 | .134 |
| H3a | .911 | .091 | .049 | .768 | .834 | .704 | .295 | .152 | .153 | .407 | .652 | .945 | .654 | .438 | .300 |
| H3b | .479 | .805 | .936 | .979 | .105 | .959 | .953 | .266 | .899 | .183 | .278 | .990 | .358 | .404 | .332 |
| H3c | .392 | .305 | .512 | .526 | .731 | .290 | .348 | .871 | .221 | .621 | .933 | .211 | .509 | .648 | .478 |
